# PSA doubling time 4.65 months as an optimal cut-off of Japanese nonmetastatic castration-resistant prostate cancer

**DOI:** 10.1038/s41598-024-65969-3

**Published:** 2024-07-03

**Authors:** Shinichi Sakamoto, Kodai Sato, Takahiro Kimura, Yoshiyuki Matsui, Yusuke Shiraishi, Kohei Hashimoto, Hideaki Miyake, Shintaro Narita, Jun Miki, Ryuji Matsumoto, Takuma Kato, Toshihiro Saito, Ryotaro Tomida, Masaki Shiota, Akira Joraku, Naoki Terada, Shigetaka Suekane, Tomoyuki Kaneko, Shuichi Tatarano, Yuko Yoshio, Takayuki Yoshino, Naotaka Nishiyama, Eiryo Kawakami, Tomohiko Ichikawa, Hiroshi Kitamura

**Affiliations:** 1 Department of Urology, Chiba University Graduate School of Medicine, 1-8-1 Inohana, Chuo-ku, Chiba, 260-8670 Japan; 2 Department of Urology, The Jikei University School of Medicine, Tokyo, Japan; 3 Department of Urology, National Cancer Center Japan, Tokyo, Japan; 4 Department of Urology, Shizuoka General Hospital, Shizuoka, Japan; 5 Department of Urology, Sapporo Medical University School of Medicine, Sapporo, Japan; 6 Division of Urology, Department of Surgery Related, Kobe University Graduate School of Medicine, Kobe, Japan; 7 Department of Urology, Akita University Graduate School of Medicine, Akita, Japan; 8 Department of Urology, The Jikei University School of Medicine, Kashiwa Hospital, Kashiwa, Japan; 9 Department of Urology, Hokkaido University Faculty of Medicine, Sapporo, Japan; 10 Department of Urology, Faculty of Medicine, Kagawa University, Takamatsu, Japan; 11 Department of Urology, Niigata Cancer Center Hospital, Niigata, Japan; 12 Department of Urology, National Hospital Organization Shikoku Cancer Center, Matsuyama, Japan; 13 Department of Urology, Graduate School of Medical Sciences, Kyushu University, Fukuoka, Japan; 14 Department of Urology, Ibaraki Prefectural Central Hospital, Ibaraki Cancer Center, Kasama, Japan; 15 Department of Urology, University of Fukui, Fukui, Japan; 16 Department of Urology, Kurume University School of Medicine, Kurume, Japan; 17 Department of Urology, Teikyo University School of Medicine, Tokyo, Japan; 18 Department of Urology, Graduate School of Medical and Dental Sciences, Kagoshima University, Kagoshima, Japan; 19 Department of Nephro-Urologic Surgery and Andrology, Mie University Graduate School of Medicine, Tsu, Japan; 20 Department of Urology, University of Tsukuba, Tsukuba, Japan; 21 Department of Urology, Faculty of Medicine, University of Toyama, Toyama, Japan; 22 Department of Artificial Intelligence Medicine, Graduate School of Medicine, Chiba University, Chiba, Japan

**Keywords:** NHT, nmCRPC, Prostate cancer, PSA doubling time, Vintage, Prostate, Outcomes research

## Abstract

A multicenter study of nonmetastatic castration-resistant prostate cancer (nmCRPC) was conducted to identify the optimal cut-off value of prostate-specific antigen (PSA) doubling time (PSADT) that correlated with the prognosis in Japanese nmCRPC. Of the 515 patients diagnosed and treated for nmCRPC at 25 participating Japanese Urological Oncology Group centers, 450 patients with complete clinical information were included. The prognostic values of clinical factors were evaluated with respect to prostate specific antigen progression-free (PFS), cancer-specific survival (CSS), and overall survival (OS). The optimal cutoff value of PSADT was identified using survival tree analysis by Python. The Median PSA and PSADT at diagnosis of nmCRPC were 3.3 ng/ml, and 5.2 months, respectively. Patients treated with novel hormonal therapy (NHT) showed significantly longer PFS (HR: hazard ratio 0.38, p < 0.0001) and PFS2 (HR 0.45, p < 0.0001) than those treated with vintage nonsteroidal antiandrogen agent (Vintage). The survival tree identified 4.65 months as the most prognostic PSADT cutoff point. Among the clinical and pathological factors PSADT of < 4.65 months remained an independent prognostic factor for OS (HR 2.96, p = 0.0003) and CSS (HR 3.66, p < 0.0001). Current data represented optimal cut-off of PSADT 4.65 months for a Japanese nmCRPC.

## Introduction

Prostate cancer is the leading cause of male death in the United States^1^. The development of castration-resistant prostate cancer (CRPC) is a key factor affecting the prognosis of patients with prostate cancer. Among localized prostate cancers, treated by radical prostatectomy and radiation therapy eventually relapse and require additional hormonal therapy. During hormonal therapy due to biochemical relapse after radical treatment for localized disease, approximately 20% of patients eventually develop castration resistance without radiographic progression and develop nonmetastatic castration-resistant prostate cancer (nmCRPC)^2–4^.

Recent phase III clinical trials such as PROSPER, SPARTAN, and ARAMIS have indicated the prognostic advantage of novel hormonal therapy (NHT) in patients with high-risk nmCRPC^5–7^. The PSA doubling time (PSADT) is associated with the development of metastasis or death in nnCRPC^8^. Based on the entry criteria of these clinical trials, a PSADT of ≤ 10 months should be the cutoff point for patients with nmCRPC who are treated with NHT. Although recent novel imaging based on prostate-specific membrane antigen ligand positron emission tomography (PSMA-PET) has indicated the presence of distant metastasis in approximately 55% of nmCRPC patients^8^, the majority of clinical trials and daily practice in this setting, are still based on conventional imaging. Thus, PSADT plays a key role in determining the treatment strategy for patients with nmCRPC. Previous phase III clinical trials have demonstrated the prognostic advantage of combined androgen deprivation therapy with bicalutamide and luteinizing hormone-releasing hormone (LH-RH) over LH-RH monotherapy in Japanese patients with locally advanced prostate cancer without metastasis^9^. Thus, Japanese patients with non-metastatic prostate cancer have traditionally been treated with vintage nonsteroidal antiandrogen agents (vintage) because of their relatively high sensitivity and low financial burden^4^.

As the treatment landscape of Japanese patients with nmCRPC is unique, the aim of this study was to conduct a multi-institutional study to examine the prognostic difference between patients who received Vintage and NHT, and to identify the optimal cut-off for PSADT.

## Results

### Patient characteristics

The demographic characteristics of the 450 patients at presentation are summarized in Table 1. The median follow-up period in the entire cohort was 33 months, and the median age at diagnosis was 71 years. The median PSA level at the time of diagnosis of nmCRPC was 3.3 ng/ml. Lymph node metastasis was positive in 17.3% of the patients. The number of patients treated with primary radical prostatectomy, radiation therapy, and androgen deprivation therapy, including Vintage/LH-RH, were 97 (22.1%), 153 (34.9%), and 188 (42.9%), respectively. There were 180 and 270 patients in the vintage and NHT groups, respectively. In the NHT group, the number of patients treated with Enzalutamide, Abiraterone, Apalutamide and Darolutamide were, 121 (44.8%), 49 (18.1%), 47 (17.4%), and 47 (17.4%), respectively; and 6 patients received docetaxel. In the Vintage group, 173 (96.1%) patients received vintage drugs, and seven (1.6%) patients received LH-RH alone. There was a significant difference between the groups in terms of PSA value (p = 0.0121) and Gleason Score (GS) ≥ 8 (p = 0.0180), with no other significant difference observed.

**Table 1:** Patient characteristics.

Variable	Overall	Vintage	NHT	P
Number	450	180	270	
Age (years)	71 (49–94)	73 (50–87)	71 (49–94)	0.1734
PSA at biopsy (ng/mL)	23 (3.2–49,992)	20.4 (3.2–87.9)	24.9 (4.7–4992)	0.1806
PSA (ng/mL)	3.3 (0.04–121.2)	2.9 (0.05–39.5)	3.9 (0.04–178.9)	0.0121*
PSADT (M)	5.26 (0.32–82.25)	5.09 (0.32–47.14)	5.32 (0.73–82.25)	0.7717
Hb (g/dL)	13.1 (8.4–16.1)	13 (9.8–16)	13.1 (8.4–17.6)	0.4625
Performance status				
PS ≥ 1	98 (23.4%)	40 (24.2%)	58 (22.8%)	0.7398
PS < 1	321 (76.6%)	125 (75.8%)	196 (77.2%)	
Unknown	31	15	16	
Biopsy Gleason score				
GS ≥ 8	272 (70.3%)	95 (63.3%)	177 (74.7%)	0.0180*
GS < 8	115 (29.7%)	55 (36.7%)	60 (25.3%)	
Unknown	63	30	33	
cT stage at biopsy				
cT ≥ 3	232	98 (60.9%)	134 (58.3%)	0.605
cT < 3	159	63 (39.1%)	96 (41.7%)	
Unknown	59	19	4	
cN stage				
cN1	72 (17.3%)	25 (15.8%)	47 (18.3%)	0.5176
cN0	343 (82.7%)	133 (84.2%)	210 (81.7%)	
Unknown	35	22	13	
Primary treatment				
Prostatectomy	97 (22.1%)	35 (20.1%)	62 (23.5%)	0.4417
Radiation therapy	153 (34.9%)	58 (33.3%)	95 (36.0%)	
Vintage・ADT	188 (42.9%)	81 (46.6%)	107 (40.5%)	
Unknown	12	6	6	
Treatment for nmCRPC				
Enzalutamide	121 (26.9%)		121 (44.8%)	
Abiraterone	49 (10.9%)		49 (18.1%)	
Apalutamide	47 (10.4%)		47 (17.4%)	
Darolutamide	47 (10.4%)		47 (17.4%)	
Docetaxel	6 (1.3%)		6 (2.2%)	
Vintage	173 (38.4%)	173 (96.1%)		
LH-RH	7 (1.6%)	7 (3.9%)		

*NHT* novel hormonal therapy, *cN1* clinical positive pelvic lymph node metastasis, *cT stage* clinical T stage, *Hb* hemoglobin, *HR* hazard ratio, *nmCRPC* nonmetastatic CRPC, *PS* performance status, *PSA* prostate-specific antigen, *PSADT* PSA doubling time, *Vintage* vintage androgen receptor antagonist.

*P < 0.05.

### Prognostic outcomes in nmCRPC patients

**Figure 1:**
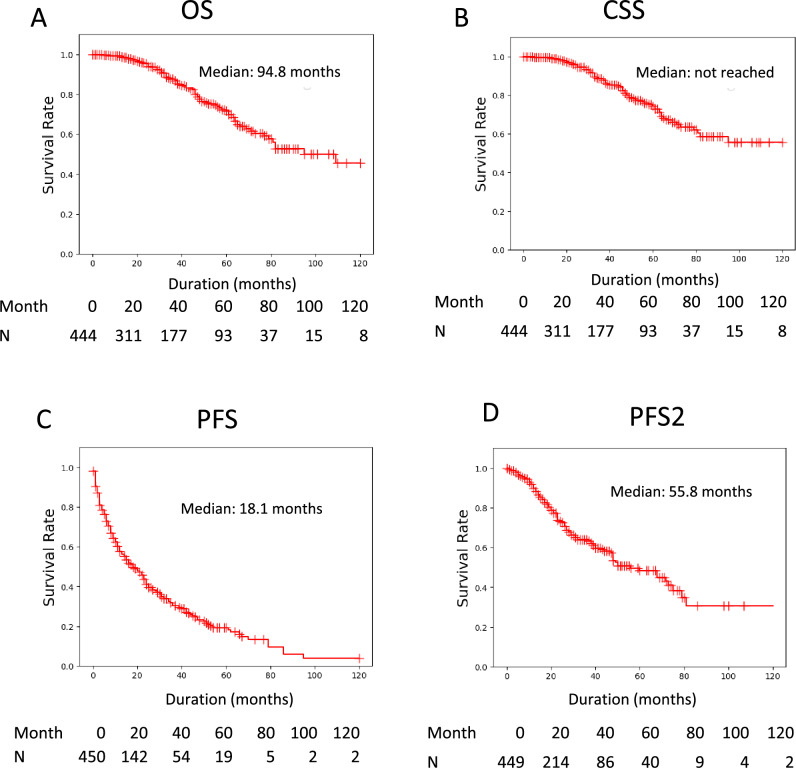
Prognosis of nmCRPC patients. (**A**) Overall survival (OS), cancer-specific survival (CSS), progression-free survival (PFS), and time to second progression or death (PFS2) were analyzed by Kaplan–Meier method.

**Figure 2:**
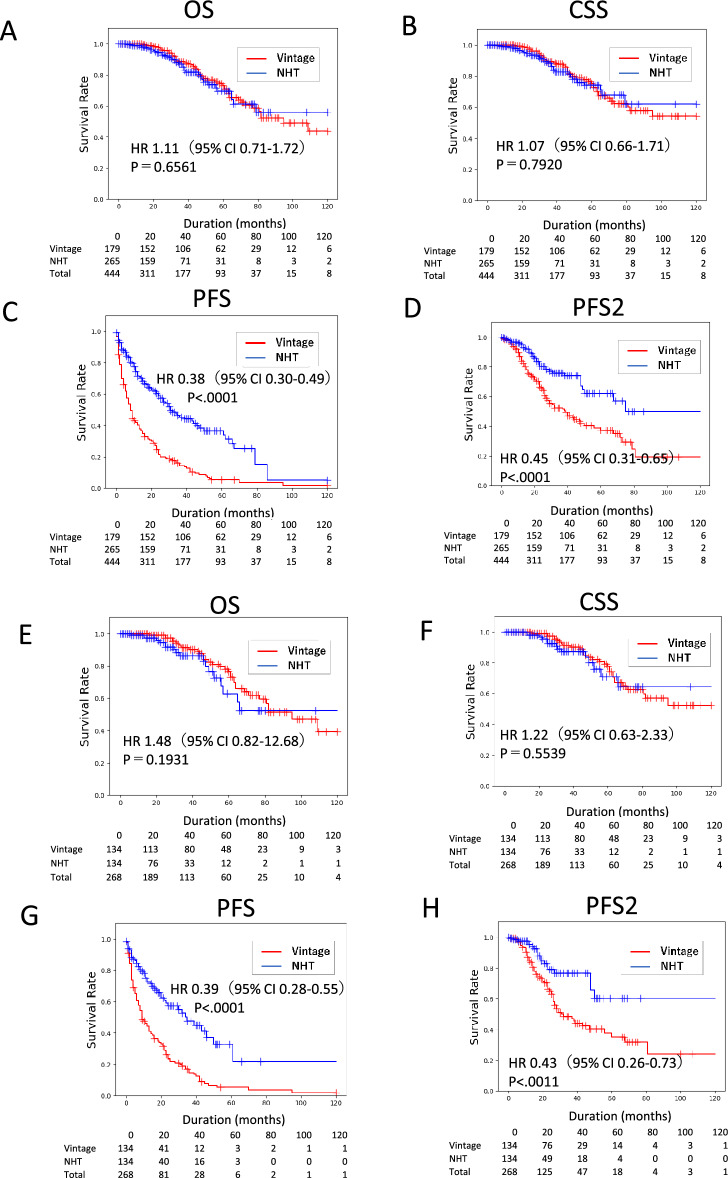
Prognostic comparison of nmCRPC patients treated by NHT and Vintage for whole cohort and propensity-score-matched pair cohort. The whole cohort of (**A**) overall survival (OS), (**B**) cancer-specific survival (CSS), (**C**) progression-free survival (PFS), and (**D**) time to second progression or death (PFS2) of nmCRPC patients treated with NHT and Vintage were analyzed using the Kaplan–Meier method. Propensity score-matched pair cohorts of (**E**) OS, (**F**) CSS, (**G**) PFS, and (**H**) PFS2 of nmCRPC patients treated with NHT and Vintage were analyzed using the Kaplan–Meier method. Statistical significance was evaluated using the log-rank test. The hazard risk (HR) was evaluated using a proportional hazard model.

In the 450 nmCRPC patients, the median OS, PFS, and PFS2 were 94.8, 18.1, and 55.8 months, respectively, whereas CSS did not reach the median (Fig. 1). Survival was significantly longer in the NHT group than in the Vintage group for PFS (Hazard Ratio: HR 0.38, p < 0. 0001) and PFS2 (HR 0.45, p < 0.0001), but not for OS (HR 1.11, p = 0.6562) or CSS (HR 1.07, p = 0.7920) (Fig. 2A–D). We conducted a propensity score-matched analysis to adjust for patient background between groups (Table S1). In the matched cohort, the prognostic trends for Vintage and NHT were similar, and significant differences were found for PFS and PFS2, but not for OS and CSS (Fig. 2E–H). To enable a comparison with previous clinical trials that adopted the entry criteria of PSADT ≤ 10 months, we performed a sub-analysis of nmCRPC patients with PSADT ≤ 10 months. Our data indicated longer PSA-PFS and OS in the control arm than in previous clinical trials of nmCRPC^6,7,10^. The prognostic trends were similar between the Vintage and NHT groups, with significant differences in PFS and PFS2, but not in OS and CSS (Fig. S1).

### Optimal cut-off value of PSADT

**Figure 3:**
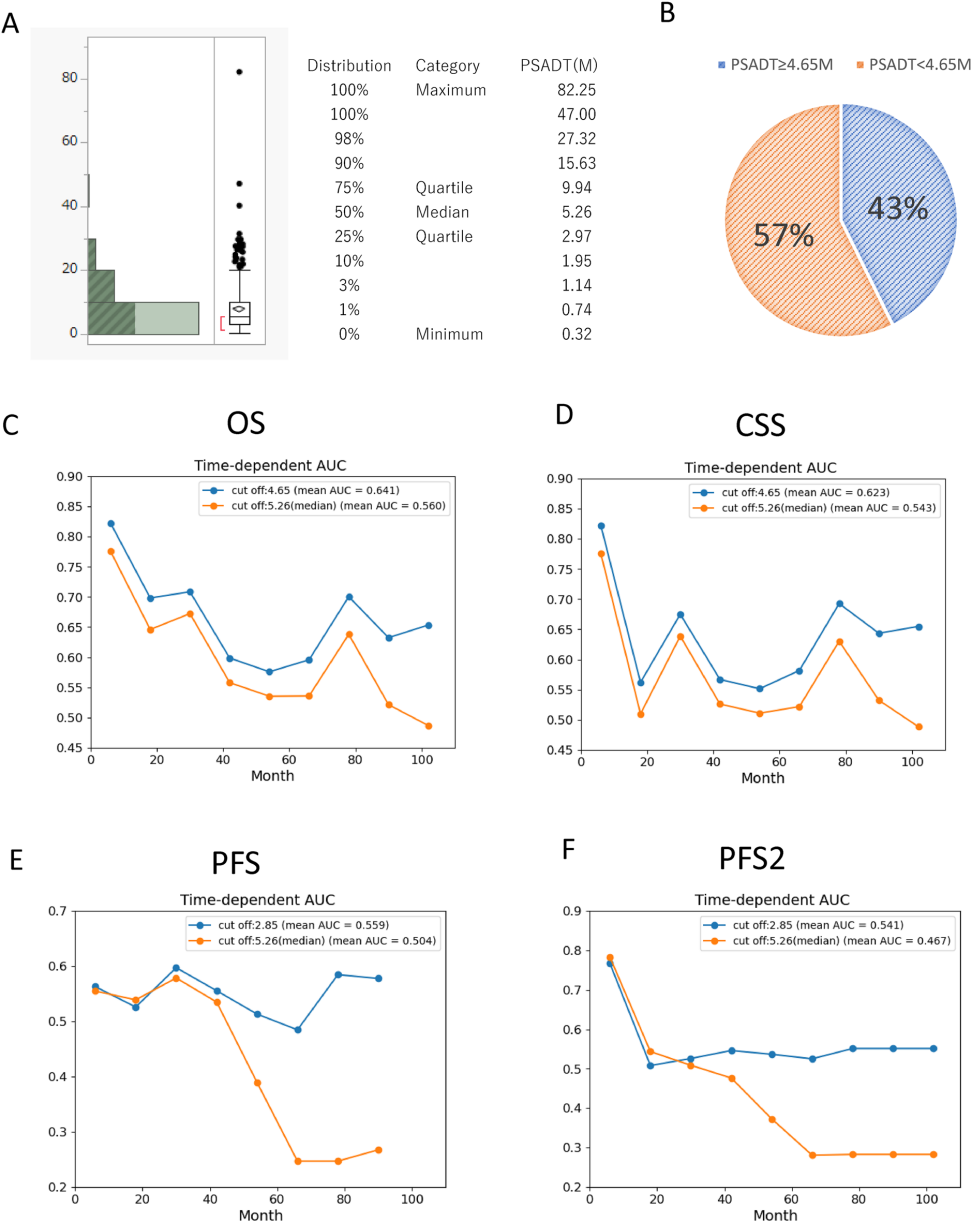
Distribution of PSA doubling time (PSADT) and the time-dependent AUC of PSADT derived by survival forest. (**A**) Distribution of PSA doubling time (PSADT). (**B**) Percentage of PSADT ≤ 4.65 months and > 4.65 months. Time-dependent AUC of PSADT 4.65 months derived by survival tree and median PSADT 5.26 months for OS (**C**) and CSS (**D**). Time-dependent AUC of PSADT 2.85 months derived by survival tree and median PSADT 5.26 months for PFS and PFS2.

To further elucidate the prognostic significance of PSADT, a survival tree was used to identify the optimal cut-off values of PSADT to distinguish between good and poor prognoses. The overall median PSADT was 5.26 months (range 0.32–82.25 months) (Fig. 3A). A survival forest was adapted. The optimal cutoff for PSADT identified was 2.85 months for PFS and 4.65 months for OS and CSS. Optimal PSADT cutoff for Vintage or NHT was presented in Fig. S2.

The proportion of patients with PSADT < 4.65 months was 43% and that with PSADT ≥ 4.65 months was 57% (Fig. 3B). Time-dependent AUC demonstrated that PSADT 4.65 months derived by survival tree for OS and CSS, consistently showed better AUC at any time point compared to the AUC of the median PSADT of 5.26 months. Similarly, PSADT 2.85 months derived by survival tree for PFS demonstrated better AUC compared to the AUC of median PSADT 5.26 months after 30 months of CRPC treatment.

**Figure 4:**
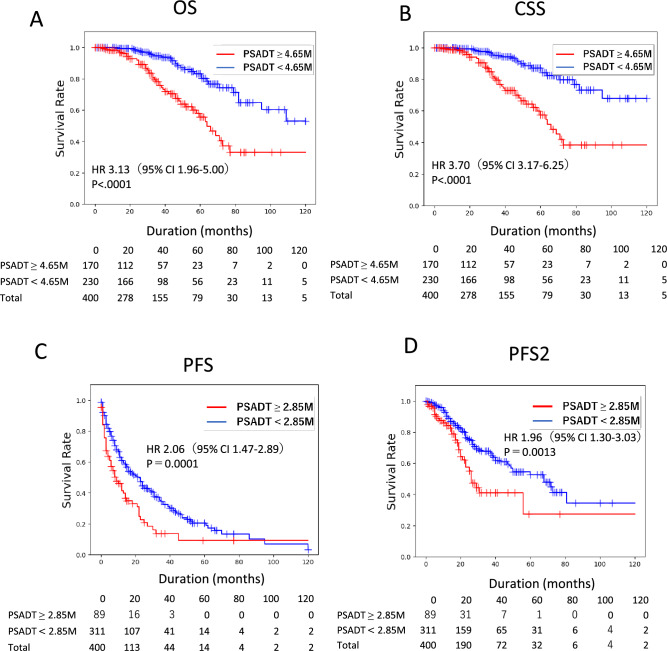
Prognostic significance of PSA doubling time (PSADT) cut-off derived by survival tree. (**A**) Overall survival (OS), and (**B**) cancer-specific survival (CSS) of nmCRPC patients with PSADT cut-offs of < 4.65 months and ≥ 4.65 months were analyzed by Kaplan–Meier method. (**C**) Progression-free survival (PFS) and (**D**) time to second progression or death (PFS2) of nmCRPC patients with PSADT cut-offs of < 2.85 months and ≥ 2.85 months were analyzed by Kaplan–Meier method. Statistical significance was evaluated by log-rank test. Hazard risk (HR) was evaluated by proportional hazard model.

Patients with PSADT < 4.65 months had significantly shorter survival than those with PSADT ≥ 4.65 months. For PFS and PFS2, patients with PSADT < 2.85 months had significantly shorter survival times than those with PSADT ≥ 2.85 months (Fig. 4).

Based on proportional hazard analysis, PSADT < 4.65 months was independently associated with OS (HR 2.96, p = 0.0003) (Table 2) and CSS (HR 3.66, p < 0.0001) (Table 3), and PSADT < 2.85 months was an independent prognostic factor for PFS (HR 1.87, p < 0.0001) (Table 4). To assess the presence of prognostic differences between NHT and Vintage among high-risk cohorts, we also performed a sub-analysis of patients with PSADT < 4.65 months. No significant differences in OS and CSS were observed between the NHT and Vintage groups (Fig. S3). A comparison between recent nmCRPC clinical trials and current data is summarized in Table 5. Among PSADT ≤ 10 months, our cohorts in the control arm showed longer overall survival (approximately 20 months) compared to the survival in the control arm of global clinical trials.

**Table 2:** Univariate and multivariate Cox proportional hazard models for OS.

Variable	Univariate analysis		Multivariate analysis	
	HR	P-value	HR	P-value
PS ≥ 1	1.91 (1.17–3.12)	0.0055*	1.10 (0.56–2.13)	0.7835
Age ≥ 71 (years)	2.60 (1.61–4.15)	< 0.0001*	2.83 (1.57–5.08)	0.0005*
cT ≥ 3	1.57 (0.95–2.57)	0.0700		
cN1 ( +)	2.52 (1.54–4.12)	0.0002*	2.47 (1.25–4.88)	0.0089*
Hb ≥ 13.1 (g/dL)	0.59 (0.36–0.95)	0.0229*	0.56 (0.32–0.98)	0.0422*
PSADT < 10 (months)	2.75 (1.66–4.56)	< 0.0001*		
PSADT < 6 (months)	2.26 (1.19–4.31)	0.0128*		
PSADT < 4.65 (months)	3.16 (1.94–5.14)	< 0.0001*	2.96 (1.65–5.32)	0.0003*
PSA ≥ 3.3 (ng/dL)	1.76 (1.12–2.74)	0.0132*	0.84 (0.47–1.51)	0.5625
NHT vs. Vintage	1.10 (0.71–1.72)	0.6562		

*NHT* novel hormonal therapy, *cN1* clinical positive pelvic lymph nodes metastasis, *cT stage* clinical T stage, *Hb* hemoglobin, *HR* hazard ratio, *OS* overall survival, *PS* performance status, *PSA* prostate specific antigen, *PSADT* PSA doubling time, *Vintage* vintage androgen receptor antagonist.

*P < 0.05.

**Table 3:** Univariate and multivariate Cox proportional hazard models for CSS.

Variable	Univariate analysis		Multivariate analysis	
	HR	P-value	HR	P-value
PS ≥ 1	1.96 (1.16–3.32)	0.0124*	1.49 (0.78–2.86)	0.2278
Age ≥ 71 (years)	2.60 (1.57–4.13)	0.0002*	3.11 (1.71–5.64)	0.0002*
cT ≥ 3	1.53 (0.91–2.58)	0.11		
cN1 (+)	2.70 (1.61–4.55)	0.0002*	2.47 (1.29–4.71)	0.0062*
Hb ≥ 13.1 (g/dL)	0.64 (0.38–1.08)	0.0946		
PSADT < 10 (months)	2.65 (1.26–5.57)	0.0102*		
PSADT < 6 (months)	2.75 (1.66–4.56)	< 0.0001*		
PSADT < 4.65 (months)	3.72 (2.18–6.37)	< 0.0001*	3.66 (2.01–6.68)	< 0.0001*
PSA ≥ 3.3 (ng/dL)	1.80 (1.11–2.91)	0.0167*	1.52 (0.85–2.72)	0.1627*
NHT vs. vintage	1.07 (0.66–1.71)	0.7920		

*NHT* novel hormonal therapy, *cN1* clinical positive pelvic lymph node metastasis, *cT stage* clinical T stage, *Hb* hemoglobin, *PS* performance status, *HR* hazard ratio, *PSA* prostate-specific antigen, *PSADT* PSA doubling time, *Vintage* vintage androgen receptor antagonist.

*P < 0.05.

**Table 4:** Univariate and multivariate Cox proportional hazard models for PFS.

Variable	Univariate analysis		Multivariate analysis	
	HR	P-value	HR	P-value
PS ≥ 1	1.27 (0.95–1.70)	0.1074		
Age ≥ 71 (years)	1.37 (1.07–1.76)	0.0139*	1.16 (0.89–1.52)	0.2616
cT ≥ 3	1.18 (0.91–1.54)	0.2100		
cN1 (+)	1.38 (1.00–1.91)	0.0498		
Hb ≥ 13.1 (g/dL)	0.86 (0.66–1.12)	0.2566		
PSADT < 10 (months)	1.42 (1.09–1.82)	0.0085*		
PSADT < 6 (months)	1.41 (1.05–1.93)	0.0249*		
PSADT < 2.85 (months)	2.06 (1.47–2.89)	< 0.0001*	2.07 (1.47–2.91)	< 0.0001*
PSA ≥ 3.3 (ng/dL)	0.99 (0.78–1.26)	0.9483		
NHT vs. vintage	0.39 (0.30–0.49)	< 0.0001*	0.38 (0.29–0.49)	< 0.0001*

*NHT* novel hormonal therapy, *cN1* clinical positive pelvic lymph node metastasis, *cT stage* clinical T stage, *Hb* hemoglobin, *HR* hazard ratio, *PS* performance status, *PFS* progression-free survival, *PSA* prostate-specific antigen, *PSADT* PSA doubling time, *Vintage* vintage androgen receptor antagonist.

*P < 0.05.

**Table 5:** Summary of clinical trials and the current study.

Entry	SPARTAN (apalutamide)	PROSPER (enzalutamide)	ARAMIS (darolutamide)	JUOG study	
	PSADT ≤ 10 months	PSADT ≤ 10 months	PSADT ≤ 10 months	PSADT ≤ 10 months	Whole cohort
Median PSADT (months)	4.4	4.5	4.5	4.3	5.3
PSA (ng/mL)	7.8	9.2	9.2	3.1	3.3
F/U periods	52	48	29	33	33
PSA-PFS (months) ARAT/cont	NR/3.7 (HR 0.06)	37.2/3.9 (HR 0.07)	NR/NR (HR 0.71)	29.4/6.0 (HR 0.34)	29.7/8.2 (HR 0.38)
OS (months) ARAT/cont	73.9/59.9 (HR 0.78)	67.0/56.3 (HR 0.73)	NR/NR (HR 0.69)	NR/76.7 months (HR 1.04)	NR/94.8 months (HR 1.11)

*NHT* novel hormonal therapy, *Cont* control, *F/U periods* follow-up periods, *HR* hazard ratio, *NR* not reached, *OS* overall survival, *PSA* prostate specific antigen, *PSADT* PSA doubling time, *PFS* progression-free survival.

## Discussion

The present results show a significant association between PSADT and prognosis in Japanese patients with nmCRPC. The survival tree identified an optimal PSADT cutoff value of 2.85 months for PFS and 4.65 months for OS and CSS. Furthermore, patients with nmCRPC treated with NHT showed prolonged PFS and PFS2 compared to those treated with Vintage. In contrast, no significant difference was observed between the two groups in terms of OS or CSS. The current data indicate the prognostic value of PSADT and the prognostic advantage of NHT over Vintage for PFS and PFS2 among Japanese patients with nmCRPC.

Previous evidence has indicated that PSA kinetics are associated with the risk of disease progression and mortality among patients with nmCRPC. Higher baseline PSA and shorter PSADT were associated with shorter time to bone metastasis-free survival (BMFS) and mortality among 201 nmCRPC patients^11^. Among 331 patients with nmCRPC, higher baseline PSA and higher PSA velocity were associated with shorter OS and shorter BMFS^12^. In the denosumab study, analysis of the placebo arm demonstrated that a PSADT of < 8 months was associated with BMFS and OS. PSADT ≤ 10 months and PSADT ≤ 6 months were associated with shortening of BMFS and OS by 3 and 7 months, respectively; however, baseline PSA was not associated with BMFS^8,13^. Although cut-off value was 4.65 montsh, the current study also demonstrated that PSADT remained an independent prognostic factor for PFS, OS, and CSS, whereas PSA level did not. PSADT appears to be a key predictor of prognosis in Japanese patients with nmCRPC.

Japan has a relatively unique history of hormonal treatment of prostate cancer. Patients were prescribed 80 mg bicalutamide, which is higher than the 50 mg prescribed in Western countries. The prognosis of prostate cancer patients treated with Vintage androgen deprivation therapy is better than that of patients in Western countries^13^. Previous phase 3, double-blind, randomized trials have demonstrated that combined androgen blockade of bicalutamide 80 mg plus an LH-RH agonist prolonged treatment failure, time to progression, and OS compared to LH-RH monotherapy in patients with locally advanced prostate cancer without metastasis^9,14^. However, no survival advantage has been observed for combined androgen blockade over LH-RH monotherapy in metastatic hormone-sensitive prostate cancer^9,14^. Therefore, the non-metastatic stage of prostate cancer has been regarded as the main target of vintage. Recent sub-analyses of global clinical trials and real-world data of Japanese nmCRPC patients have also demonstrated the prognostic significance of NHT in terms of PFS and metastasis-free survival^10,15^, but its effect on overall survival has not been documented. The present study demonstrated the outcomes of OS and CSS for the first time in a large Japanese population with nmCRPC. Our data indicate the advantages of NHT over Vintage for PFS and PFS2, but not for OS and CSS. In the present study, the median PFS and OS in the non-NHT group (Vintage) were 6.0 and 76.7 months, respectively, among patients with PSADT ≤ 10 months. When compared with previous global clinical trials, the current data show an increase of 2 months in PFS and 20 months in OS^5–7^. In this study, median follow-up period was 33 months, which was shorter than the follow-up period in the SPARTAN study (Apalutamide: 52 months), PROSPER study (Enzalutamide: 48 months) and comparable to the ARAMIS study (Darolutamide: 29 months). Further follow-up is required to objectively assess the long-term outcomes in patients with nmCRPC. We are currently preparing follow-up study of nmCRPC by Japanese Urological Oncology Group (JUOG).

Survival tree has been applied to the treatment of various cancers^16–18^. Compared with conventional statistical analysis, a survival tree can handle greater amounts of data and comprehend the rules and patterns behind the data^17,18^. In the field of prostate cancer, radiographic images and diagnostic accuracy have been examined using machine learning and Python^19,20^; however, the prognostic factors of localized and metastatic prostate cancer, especially during androgen deprivation therapy, have not been well studied. To optimize the prognostic cutoff value of PSADT, we used a survival tree by Python. We identified a PSADT of 2.35 months for PFS and 4.65 months for OS/CSS. Among all clinical factors, including baseline PSA level, the cutoff value of PSADT was the only independent prognostic factor. Although PSADT < 4.65 months was identified as an unfavorable prognostic factor for nmCRPC, no significant difference in OS/CSS was observed between NHT and Vintage, even within this group. A recent study reported that PSMA-PET identified nearly 55% of metastases among patients with nmCRPC who were diagnosed using conventional imaging^8^. Metastatic-directed therapy may have a further prognostic advantage among high-risk nmCRPC patients^21^.

The present study has several limitations. First, we conducted a retrospective analysis and included only a limited number of patients. Second, metastasis-free survival could not be assessed due to heterogeneity and a lack of consensus in identifying radiographic progression in a real-world setting. Third, the detailed information related to the types of salvage therapies, after the recurrence of primary treatment, such as application of salvage radiation therapy after radical prostatectomy, were not assessed in this study. Fourth, the median follow-up was 32.7 months, which limits the precise assessment of long-term outcomes in patients with prostate cancer. Further follow-up analysis of currently registered patients is in progress. In conclusion, PSADT is significantly associated with the prognosis of Japanese patients with nmCRPC. In particular, a PSADT cut-off of 4.65 months may be used to identify the poor prognosis group and personalize treatment strategies. Further follow-up will elucidate the long-term outcomes of Japanese nmCRPC.

## Methods

### Patients and clinical variables

This retrospective study analyzed the data of 450 of 515 patients who were treated for nmCRPC collected from 25 hospitals in collaboration with the Japanese Urological Oncology Group (JUOG). Sixty-five cases were excluded from the analysis due to duplicate data, missing treatment information, or missing recurrence information. The primary endpoint was overall survival (OS), and the secondary endpoints were cancer-specific survival (CSS), PSA level, progression-free survival (PFS), and time to second progression or death (PFS2)^5^. Patients were subdivided into Vintage and NHT groups. The Vintage group included patients who received bicalutamide, flutamide, LH-RH therapy, and surgical castration. The NHT group included patients who received abiraterone, apalutamide, enzalutamide, or darolutamide. Seven patients treated with docetaxel were included in the NHT group. Progression was determined on the basis of PSA progression, radiographic progression, or death. PSA progression was determined based on the definition of PCWG3^22,23^.

### Statistical analysis

Univariate and multivariate Cox proportional models and the Kaplan–Meier method were used for predictive analyses. The log-rank test was used for the statistical comparison of groups using the Kaplan–Meier method. Fisher’s exact test was used to analyze the association between Vintage and NHT groups. P-values were set at significance levels of ≤ 0.05 and marginal significance levels of ≤ 0.10. Statistical computations were performed using the JMP Pro 15 software (SAS Institute, Cary, NC, USA). Propensity score-matched analysis was performed based on factors including age, PSA, and Gleason Score.

### Determination of optimal PSADT using survival tree

The optimal cutoff value for PSADT was identified using a survival tree, as described previously^23^. Briefly, a survival tree predicts the cumulative hazard function after considering survival time and censoring data. It calculates the case hazard function by majority voting over decision trees that predict survival. The threshold value of each node feature amount was calculated to maximize the difference in hazard between cases. We adopted the value of PSADT calculated in this manner as the threshold. The time-dependent area under the curve (AUC) was compared between median cut-off and the identified cut-off of PSADT.

### Ethical approval

This study was approved by the Institutional Review Board (IRB) of Chiba University Hospital (approval No. 4221) and the regional medical research review boards of all 24 hospitals participating in JUOG. Informed consent was waived by IRB (Institutional Review Board of Chiba University Hospital) due to the retrospective nature of the study. The present study was conducted in accordance with ethical standards that promote and ensure respect and integrity for all human subjects and the Declaration of Helsinki. All studies were performed in accordance with relevant named guidelines and regulations.

### Supplementary Information

Supplementary Information.

## Data Availability

The data that support the findings of this study are available from the Japanese Urological Oncology Group (JUOG), but restrictions apply to the availability of these data, which were used under license for the current study and so are not publicly available. The data are, however, available from the authors upon reasonable request and with the permission of the Japanese Urological Oncology Group (JUOG). The contact should be made to a corresponding author: Shinichi Sakamoto, E-mail: rbatbat1@chiba-u.jp, Chiba University Graduate School of Medicine, 1-8-1 Inohana, Chuou-ku, Chiba, Japan.

## Abbreviations

AR: Androgen receptor
NHT: Novel hormonal therapy
CRPC: Castration-resistant prostate cancer
nmCRPC: Nonmetastatic castration-resistant prostate cancer
PSA: Prostate-specific antigen
PSADT: PSA doubling time
PS match: Propensity score match
Vintage: Vintage nonsteroidal antiandrogen agent
OS: Overall survival
PFS: Progression-free survival
CSS: Cancer-specific survival
LH-RH: Luteinizing hormone-releasing hormone
