# Supplementary material for: PSA doubling time 4.65 months as an optimal cut-off of Japanese nonmetastatic castration-resistant prostate cancer
Source: Sci Rep. 2024 Jul 3;14:15307. doi: 10.1038/s41598-024-65969-3 (PMC11222484; doi:10.1038/s41598-024-65969-3)

**Supporting information**

| Table S1. Patient characteristics in propensity-score-matched cohort | | | |
| --- | --- | --- | --- |
| Variable | Vintage | NHT | P |
| Number | 134 | 134 |  |
| Age (years)** | 71 | 72 | 0.448 |
| PS ≥ 1 | 28 (22.2%) | 27 (21.1%) | 0.827 |
| PSA at biopsy (ng/mL) | 20.1 | 23.4 | 0.784 |
| PSA (ng/mL)** | 2.8 | 3.8 | 0.631 |
| PSADT (M) | 5.0 | 5.4 | 0.911 |
| GS ≥ 8** | 89 (66.4%) | 95 (70.9%) | 0.429 |
| Hb (g/dL) | 13.1 | 13.2 | 0.798 |
| cN1 | 16 (14.0%) | 19 (14.8%） | 0.858 |
| Primary treatment |  |  |  |
| Prostatectomy | 29 (22.0%） | 29 (22.1%) | 0.8885 |
| Radiation therapy | 46 (34.9%) | 49 (37.4%) |  |
| Vintage | 57 (43.2%) | 53 (40.5%) |  |
| NHT; novel hormonal therapy, cN1; clinical positive pelvic lymph node metastasis, DOC; docetaxel, Hb; hemoglobin, PS; performance status, PSA; prostate specific antigen, PSADT; PSA doubling time, PS matched; propensity-score-matched, Vintage; Vintage androgen receptor antagonist. *P <0.05, ** factors used for the propensity score match | | | |


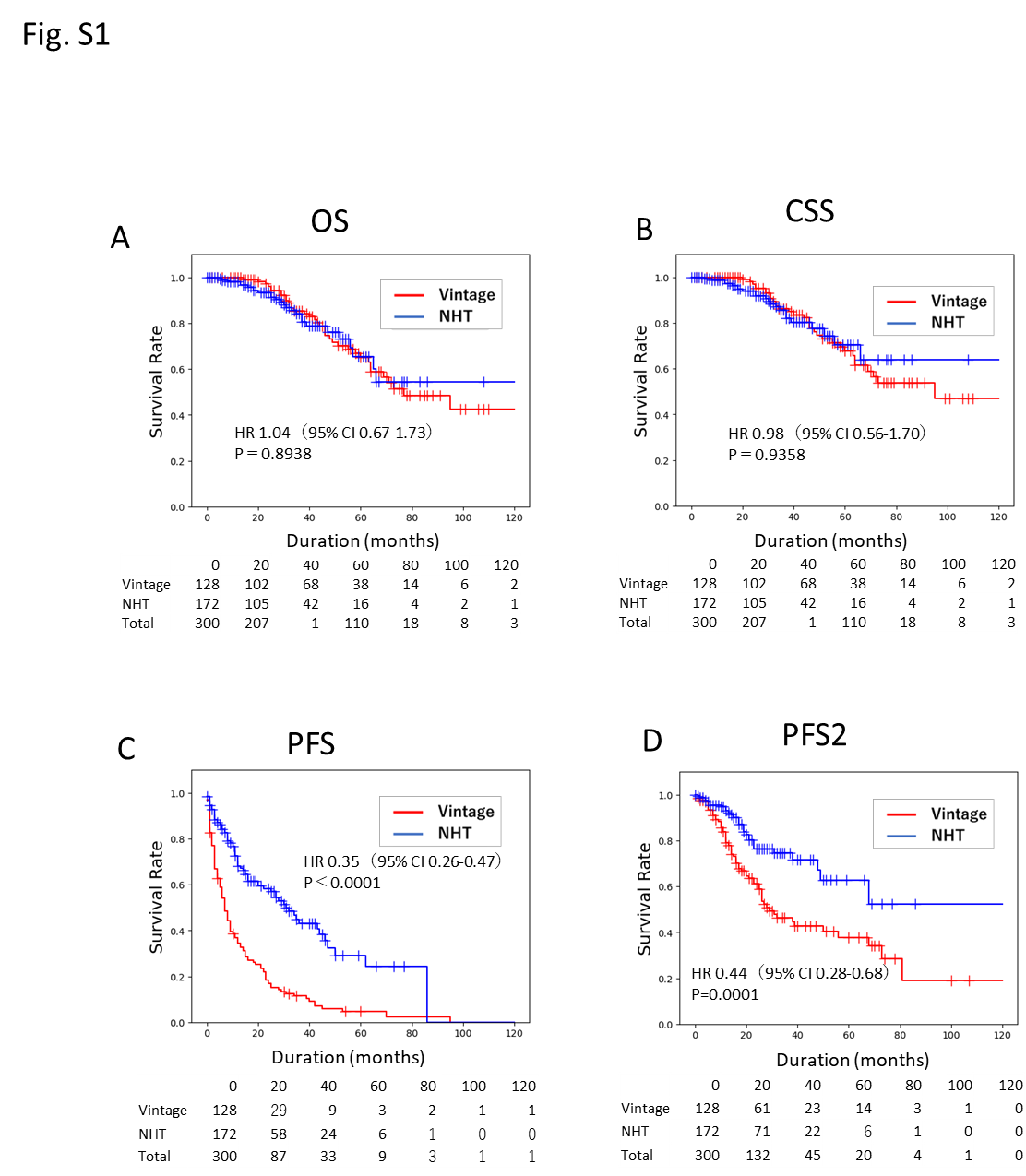


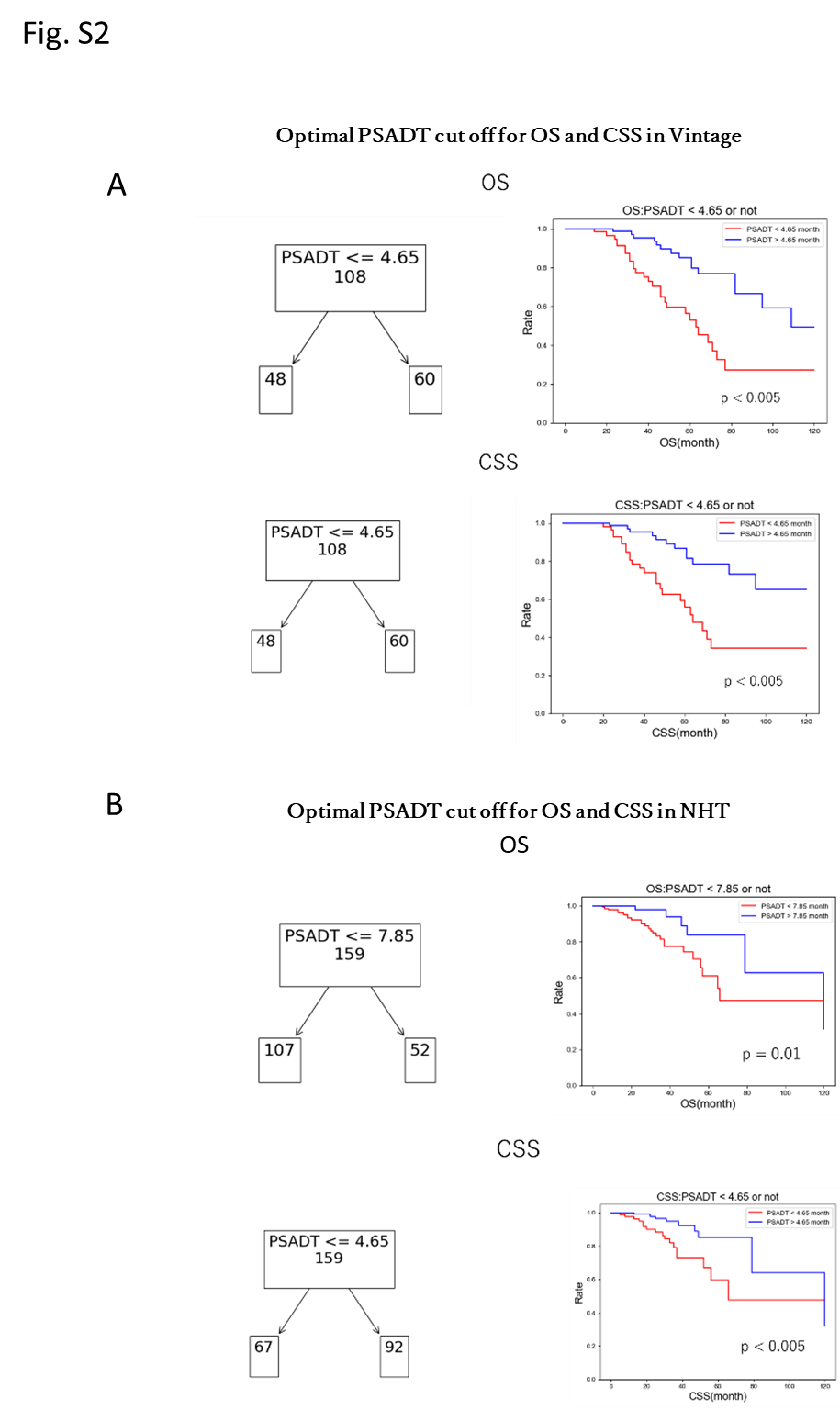


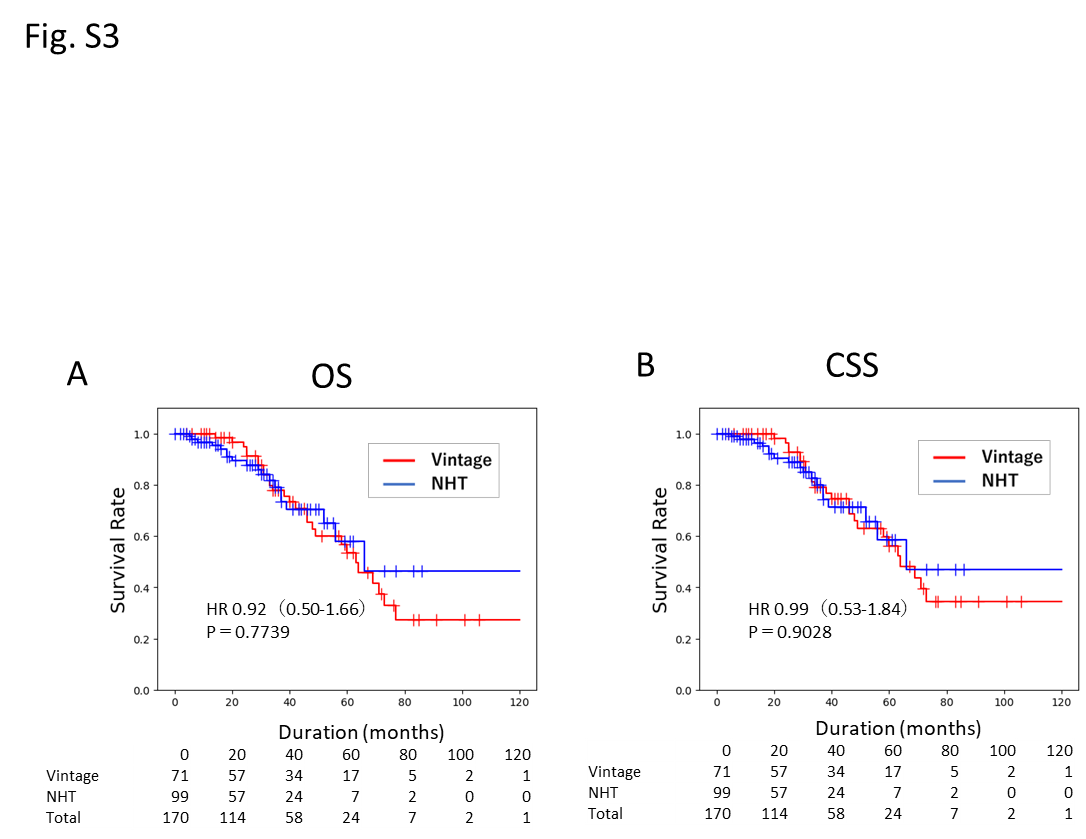

Supplement: Supplementary file 1 — Supplementary Information. [file 41598_2024_65969_MOESM1_ESM.docx]
